# A case-driven hypothesis for multi-stage crack growth mechanism in fourth-generation ceramic head fracture

**DOI:** 10.1186/s13018-022-03190-6

**Published:** 2022-06-03

**Authors:** Stefano Lucchini, Massimiliano Baleani, Federico Giardina, Andrea Martelli, Francesco Castagnini, Barbara Bordini, Francesco Traina

**Affiliations:** 1 IRCCS Istituto Ortopedico Rizzoli, Ortopedia-Traumatologia e Chirurgia Protesica e dei Reimpianti d’Anca e Ginocchio, Bologna, Italy; 2 IRCCS Istituto Ortopedico Rizzoli, Laboratorio di Tecnologia Medica, Bologna, Italy

**Keywords:** Hip prosthesis, Ceramic bearing, Ceramic head fracture, Fracture rate, Mode of failure

## Abstract

**Background:**

Ceramic bearings are used in total hip arthroplasty due to their excellent wear behaviour and biocompatibility. The major concern related to their use is material brittleness, which significantly impacts on the risk of fracture of ceramic components. Fracture toughness improvement has contributed to the decrease in fracture rate, at least of the prosthetic head. However, the root cause behind these rare events is not fully understood. This study evaluated head fracture occurrence in a sizeable cohort of patients with fourth-generation ceramic-on-ceramic implants and described the circumstances reported by patients in the rare cases of head fracture.

**Methods:**

The clinical survivorship of 29,495 hip prostheses, with fourth-generation ceramic bearings, was determined using data from a joint replacement registry. The average follow-up period was 5.2 years (range 0.1–15.6). Retrieval analysis was performed in one case for which the ceramic components were available.

**Results:**

Clinical outcomes confirmed the extremely low fracture rate of fourth-generation ceramic heads: only two out of 29,495 heads fractured. The two fractures, both involving 36 mm heads, occurred without a concurrent or previous remarkable trauma. Considering the feature of the fractured head, a multi-stage crack growth mechanism has been hypothesized to occur following damage at the head–neck taper interface.

**Conclusions:**

Surgeons must continue to pay attention to the assembly of the femoral head: achieving a proper head seating on a clean taper is a prerequisite to decrease the risk of occurrence of any damage process within head–neck junction, which may cause high stress concentration at the contact surface, promoting crack nucleation and propagation even in toughened ceramics.

## Background

Total hip arthroplasty (THA) is a successful surgical procedure consisting in the replacement of the coxal joint with prosthetic components. To date, the most widely used bearing couples are metal-on-polyethylene, ceramic-on-polyethylene and ceramic-on-ceramic.

Ceramic-on-ceramic (CoC) bearings have been introduced trying to minimize the risks associated with metal and polyethylene wear debris, such as particle disease, osteolysis and aseptic loosening of prosthetic’s components [1–3]. Although the choice of ceramic bearings partially resolved the aforementioned concerns due to the outstanding tribological properties, this solution left room for other specific complications such as articular noise or even component—head or liner—fracture [3–5].

During the past years, a great effort has been made to improve alumina ceramic strength and toughness with the aim of reducing the risk of component fractures. Improvements of mechanical properties have been achieved over years both improving manufacturing process, with beneficial effects on alumina microstructure, and incorporating other elements, mainly zirconia, into the microstructure [6]. These improvements in mechanical properties, also associated with a trend to use larger femoral heads, decreased the incidence of ceramic component fractures, at least of the fourth-generation ceramic heads (Biolox® Delta). Indeed, studies of clinical outcomes reported an extremely low fracture rate of the fourth-generation ceramic heads (range 0.001–0.009%) [5, 7].

Surprisingly, clinical reports showed that head fractures have occurred in unexpected circumstances, i.e. not associated with previous traumatic events [8–11]. These clinical outcomes suggest the occurrence of a more complex failure mode, other than failure related to concurrent or previous remarkable trauma associated with impact loading of the prosthetic joint.

The aim of this paper is twofold:

- to evaluate the fracture occurrence of fourth-generation ceramic heads in a sizeable cohort of patients with a CoC implant, using an arthroplasty regional registry;
- to describe the circumstances reported by patients in the rare case of head fracture, eventually associated with a retrieval analysis of the head when available.

## Methods

This study was approved by a review board (485/2020/Oss/IOR). Patient data were retrieved from the Register of Orthopaedic Prosthetic Implant (RIPO) of Emilia-Romagna region. We extracted all patients meeting the following criteria:

- all patients living in the Emilia-Romagna region, to reduce bias due to loss to follow-up;
- all patients who underwent a primary hip replacement between January 2002 and December 2019, the longest period for which clinical outcomes are available;
- all patients who received implants including a Biolox® Delta head and Biolox® Delta liner, i.e. the fourth-generation CoC bearings;
- all patients regardless of indication for hip replacement to generalize the outcomes.

All the cases of head fracture were clinically and radiographically analysed to describe the demographics, implant-related features, radiographic components positioning and the history of presentation of the symptoms. Retrieval analysis was also performed in one case in which the retrieved components—i.e. the ceramic liner, the ceramic head and the 12/14 male taper—were available.

## Results

A total of 26,401 patients met the inclusions criteria (Table 1). However, 3094 patients underwent bilateral hip replacement. Therefore, the total number of fourth-generation CoC bearings implanted in the aforementioned period was 29,495. Two out of 29,495 heads fractured leading to an overall incidence of 0.007%.

**Table 1:** Patient demographics

Number of patients	26,401
Women	56.7%
Age at surgery, mean (range)	65.8 (11–96) yrs
Weight at surgery, mean (range)	75.9 (30–178) kg
BMI at surgery	
Underweight (< 19 kg/m^2^)	1.1%
Normal (19–24.9 kg/m^2^)	31.2%
Overweight (25–29.9 kg/m^2^)	45.2%
Obese (≥ 30 kg/m^2^)	22.5%
Indication for hip replacement	Primary osteoarthritis 72.8%
	Femoral neck fracture sequelae 12.7%
	Sequelae of development dysplasia 7.5%
	Idiopathic femoral head necrosis 5.4%
	Rheumatic Arthritis 0.7%
	Other 0.9%
Follow-up, mean (range)	5.2 (0.1–15.6) yrs

### Case 1

A 67-year-old male (height 183 cm, weight 90 kg, BMI 26.9 kg/m2) underwent a THA for primary osteoarthritis of the right hip in 2008 at our Institute. Using a standard lateral approach, a cementless hemispherical Ti-alloy cup (56 mm Fixa, Adler Ortho, Milan, Italy) assembled with a tapered ceramic liner (36 mm Biolox® Delta, Ceramtec, Plochingen, Germany) was implanted. A cementless Ti-alloy anatomical stem (size 6 Apta, Adler Ortho, Milan, Italy) was press-fitted into the medullary canal. A Ti-alloy modular neck in varus configuration (0A Modula, Adler Ortho, Milan, Italy) was tightly assembled to the stem. Finally, a ceramic head (36-mm-long-neck Biolox® Delta, Ceramtec, Plochingen, Germany) was attached to the 12/14 male taper of the neck. Components’ positioning was analysed: cup abduction: 39°; centre of rotation height: 15 mm; centre of rotation medialization: 32 mm; femoral offset: 36 mm; and leg length discrepancy: + 4 mm. The patient had no complain and was satisfied with the replaced hip.

**Fig. 1:**
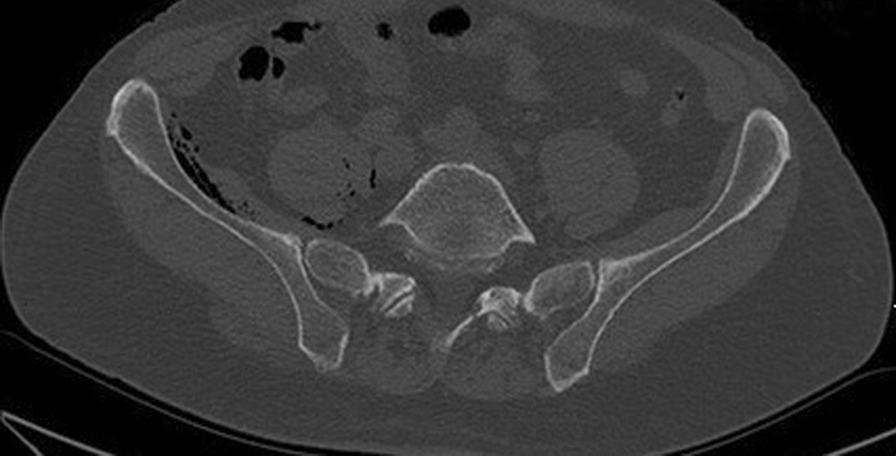
An axial slice of a CT showed the gas flap between ilium and the iliopsoas muscle in the right hip

In 2013, the patient came back to our emergency room for right hip pain, started after a simple torsional movement of the lower limb: the patient described a slight internal rotation performed while standing, with the hip flexed of about 10 degrees, in order to turn his torso. Standard X-ray evaluation showed a well-positioned and well-integrated THA. CT-scan showed a gas flap between the ilium and the iliopsoas muscle (Fig. 1), which was primarily referred to an infection sustained by gas-producing bacteria (CRP 7.6 mg/dl, normal value < 0.5 mg/dl; ESR 54 mm/h, normal value < 20 mm/h).

**Fig. 2:**
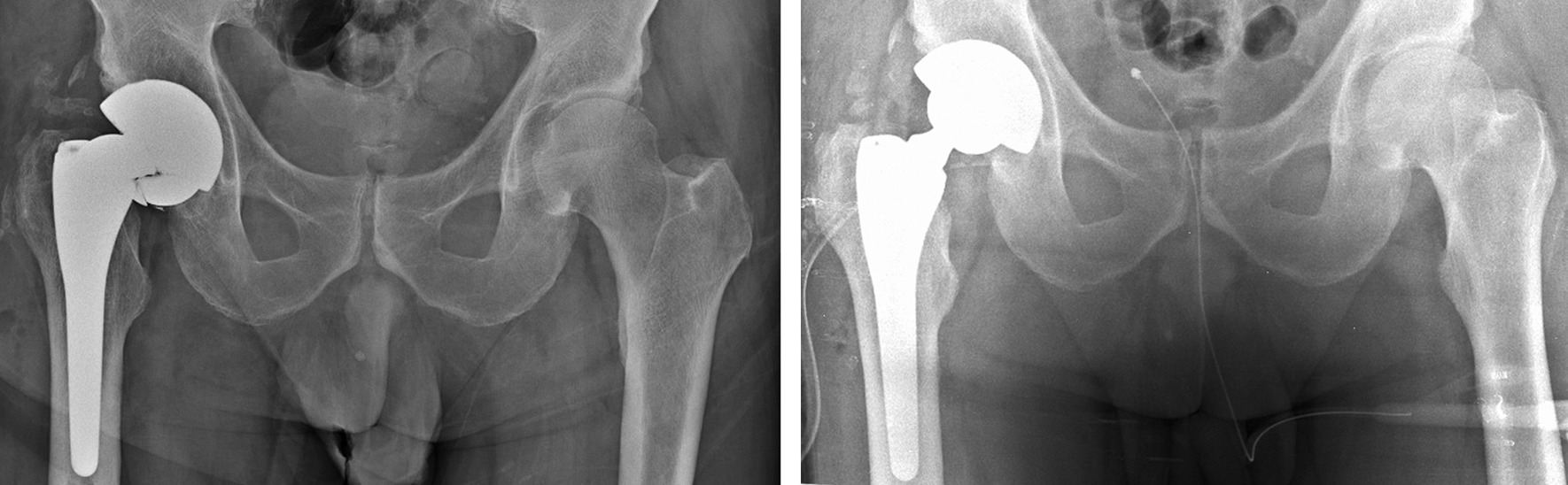
Left: preoperative X-ray showing mechanical failure of ceramic femoral head. Right: postoperative X-ray after components replacement

After two days, the patient returned to our emergency room: he complained of pain and could not weight-bear. The patient reported that he perceived an audible “pop” while walking followed by sudden pain in the prosthetic hip. Due to pain, he sustained a direct trauma on his right hip. The patient had no history of falls or trauma in the 12 months prior to femoral head fracture. Pelvis X-ray showed mechanical failure of the femoral head (Fig. 2). Revision surgery was performed replacing the fractured ceramic head, the ceramic liner and the modular neck with new identical components (Fig. 2). No signs of metallosis were observed near the implant intraoperatively at gross examination. Prosthetic joint infection was excluded by peri-prosthetic tissue culturing. The patient had a complete functional recovery, and no further complication occurred at 8.1 years of follow-up.

**Fig. 3:**
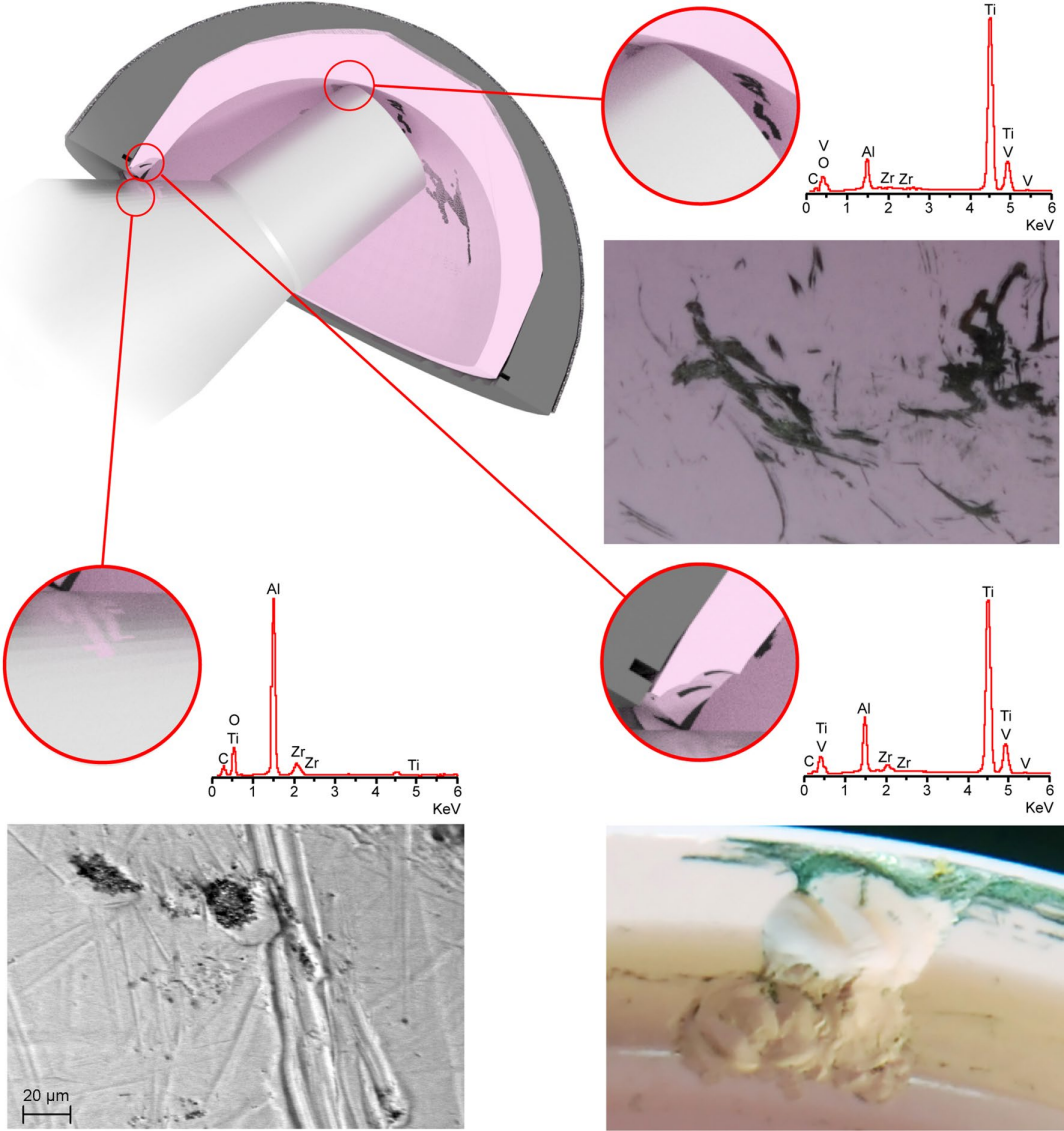
Top-left: scheme showing contact points between neck taper and ceramic liner after ceramic head fracture. Top-right: metal transfer due to impingement between taper tip and ceramic liner. EDX spectrum revealed that smears are made of Ti-alloy. Bottom-left: scratches found on the lateral side of the modular neck. EDX spectrum revealed small ceramic particles embedded into the metal. Bottom-right: chipping of the ceramic liner. EDX spectrum revealed that smears and particles on the fracture surface are made of Ti-alloy

**Fig. 4:**
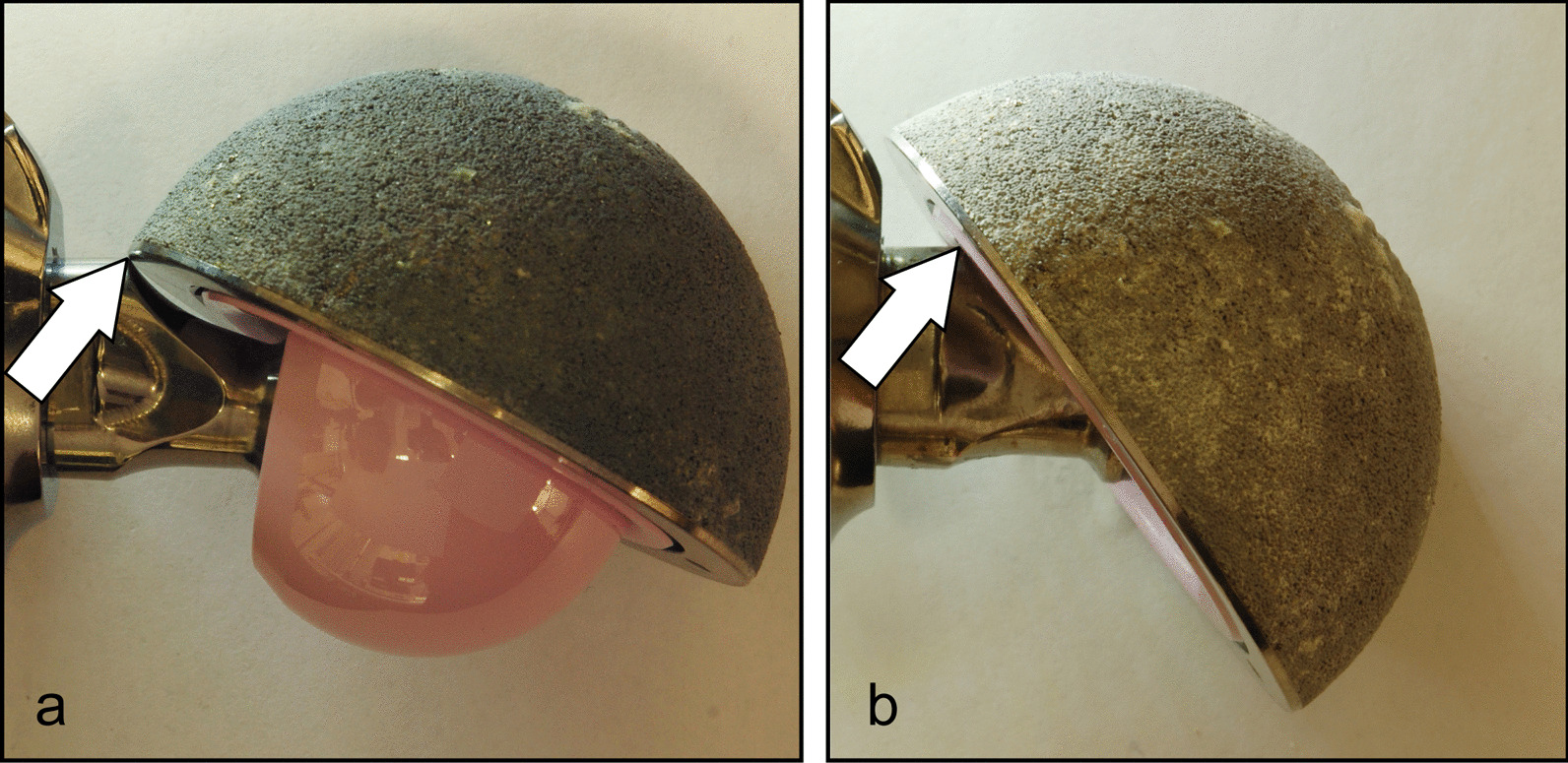
**a** Impingement between the acetabular cup and the neck with the intact ceramic head. The lateral side of the neck impinges on the metal shell (white arrow). **b** Impingement between the acetabular cup and the neck without the ceramic head. The lateral side of the neck impinges on the ceramic liner (white arrow)

**Fig. 5:**
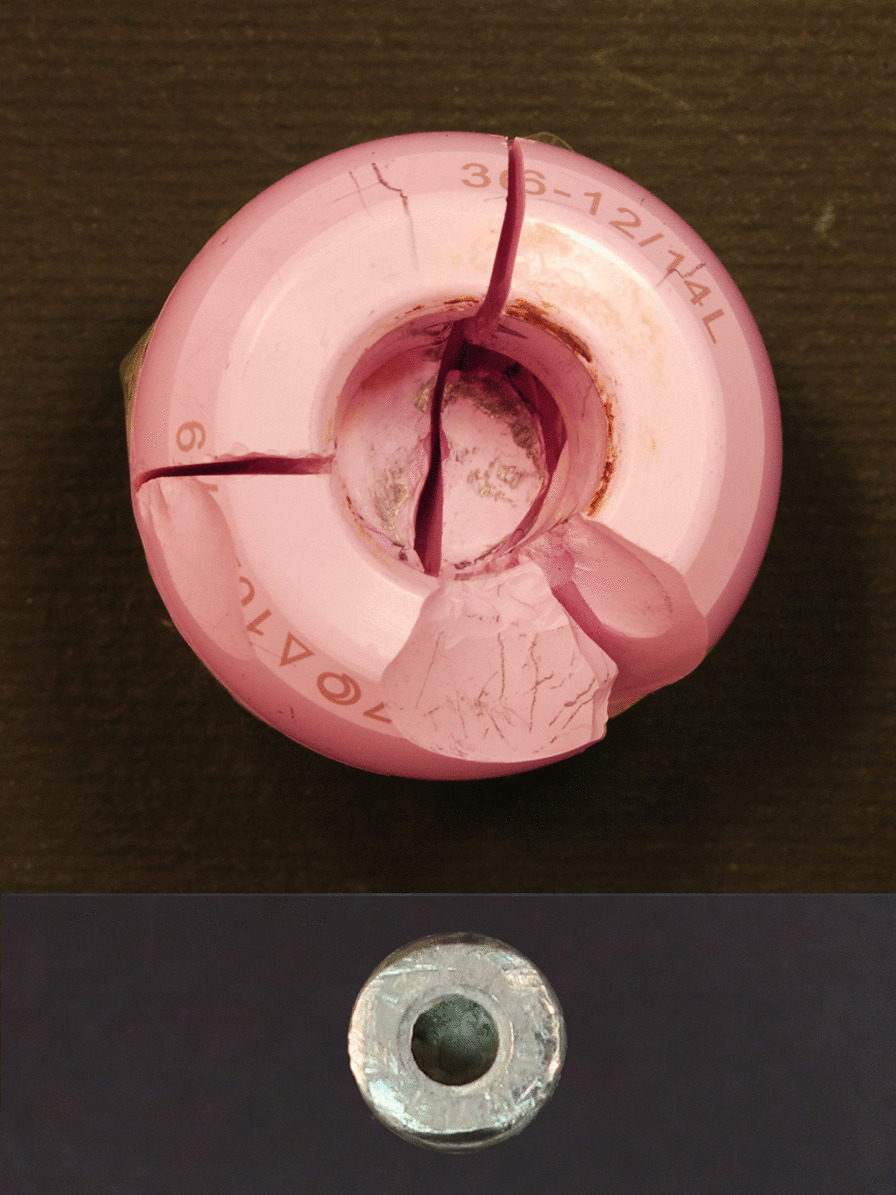
Top: Main fragments of the fractured ceramic head (caudal view). Black stains are visible at the top of the head bore. Bottom: 12/14 male taper (cranial view). The taper cross section is irregular in shape and smaller in size than that of a new taper. The top of the taper is damaged

The retrieved ceramic liner, head and modular neck underwent macroscopic and microscopic examination. The liner showed some dark smears on the inner surface (Fig. 3). Scanning electron microscope (SEM) and energy-dispersive X-ray (EDX) analysis revealed the presence of titanium (Fig. 3). Metal transfer occurred due to impingement between taper tip and ceramic liner following head fracture. Small chipping was also found on the edge of the ceramic liner (Fig. 3) likely due to impingement with the modular neck occurred during lateral fall. Indeed, SEM–EDX analysis revealed the presence of titanium closer to the chip fracture (Fig. 3). Additionally, the modular neck showed light scratches and small ceramic particles embedded into Ti-alloy where lateral neck surface can match the liner rim when the neck is fully inserted into the rim (Fig. 3). This condition can occur only following head removal or fracture (Fig. 4). Black stains were also visible at the top of the head bore due to impact with the neck taper (Fig. 5). Machining grooves were no more visible on the 12/14 male taper, and the conical surface appeared worn and irregular in shape. The head showed multiple cracks originated at different sites of the head bore surface, their interaction determining the shape and number of fragments. It was impossible to identify the origin of the very first critical crack due to head fragmentation and material loss.

**Fig. 6:**
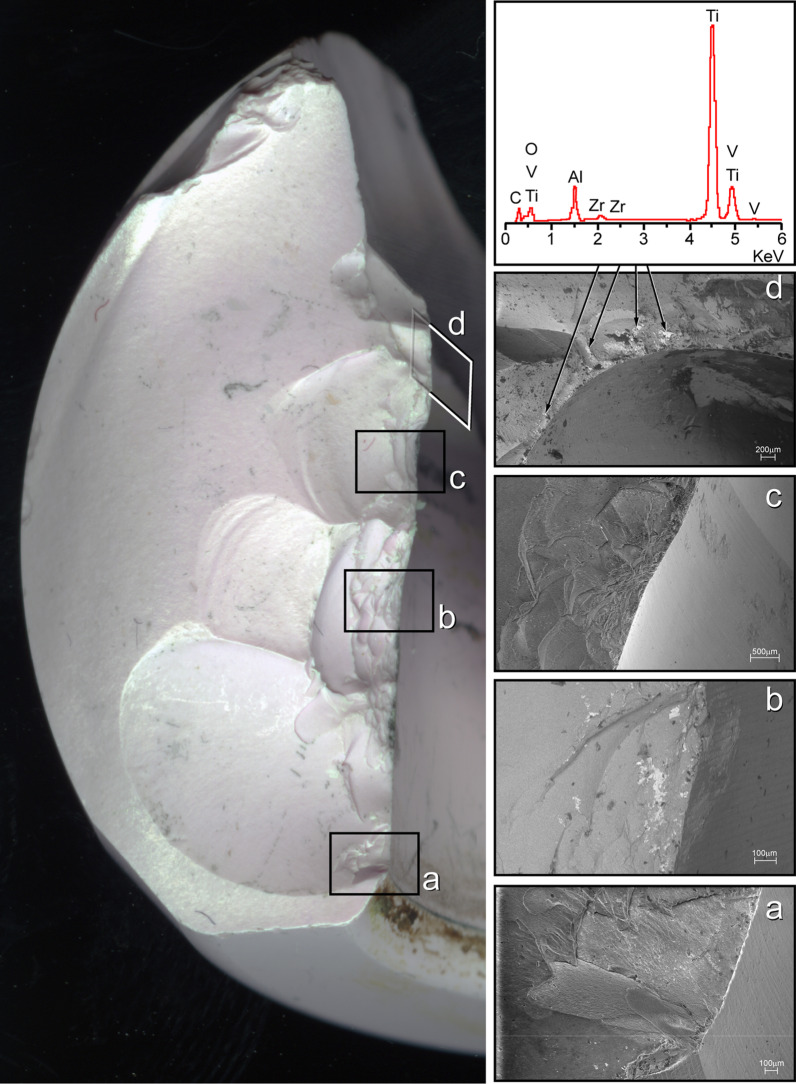
Left: fracture surface of the main fragment. **a** Possible origin of the ceramic fracture. **b** Secondary crack originated at the level of the proximal end of the neck taper. **c** Secondary crack originated at the level of the proximal end of the neck taper following to the first shift of the taper. **d** Secondary crack originated at the level of the proximal end of the neck taper following to the second shift of the taper (note: the tip of the taper impacts to the top of the head bore). EDX spectrum revealed that particles on the fracture surface are made of Ti-alloy

However, fracture surface features suggest that fracture originated at the lower level of the bore–taper contact, radiating outwards and upwards (Fig. 6). Several secondary cracks were found at the level of the proximal end of the neck taper, which gradually shifted towards the top of the head bore (Fig. 6). Indeed, secondary cracks were found at the corner of the head bore (Fig. 6). Chipping, associated with the transfer of Ti-alloy, was found on the bore surface near secondary fracture origins. SEM–EDX analysis revealed the presence of titanium particles spread on or scattered over the fracture surface. Titanium particles were found on smooth and semielliptical regions of the fracture surfaces (Fig. 6).

### Case 2

A 50-year-old (height 178 cm, weight 85 kg, BMI 26.8 kg/m2) male came at our Institute, in 2007, for primary right hip arthritis. Total hip arthroplasty was performed. Using a standard lateral approach, a cementless hemispherical Ti-alloy cup (60 mm Fixa, Adler Ortho, Milan, Italy) assembled with a tapered ceramic liner (36 mm Biolox® Delta, Ceramtec, Plochingen, Germany) was implanted using press-fit technique. A cementless Ti-alloy anatomical stem (size 5 Apta, Adler Ortho, Milan, Italy) was press-fitted into the medullary canal. A Ti-alloy modular neck with increased offset (0C Modula, Adler Ortho, Milan, Italy) was tightly assembled to the stem. Finally, a ceramic head (36 mm medium-neck Biolox® Delta, Ceramtec, Plochingen, Germany) was attached to the 12/14 male taper of the neck. Components’ positioning was analysed: cup abduction: 31°; centre of rotation height: 20 mm; centre of rotation medialization: 34 mm; femoral offset: 47 mm; and leg length discrepancy: + 12 mm. The patient had no complain and referred satisfaction with the replaced hip.

**Fig. 7:**
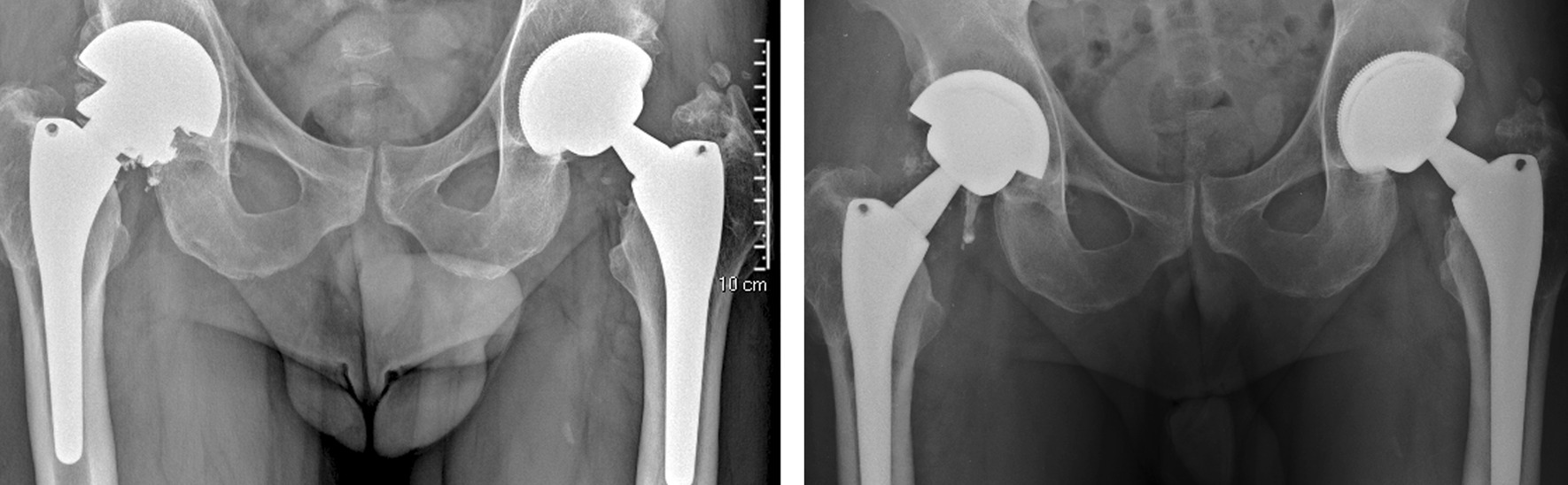
Left: preoperative X-ray showing mechanical failure of ceramic femoral head. Right: postoperative X-ray after components replacement

In 2016, the patient felt a sudden right hip pain during a simple torsional movement, described by the patient as a minimal external rotation with extended hip during stance gait phase. For the next three weeks, the patient performed his daily activities. Then, the patient underwent medical examination in another hospital following acute worsening of pain in the replaced hip joint with inability to weight bearing. The patient had no history of falls or trauma in the 12 months prior to femoral head fracture. Pelvis X-ray showed mechanical failure of the femoral head (Fig. 7), and the patient underwent revision surgery. Multiple fragments of ceramic head were removed, and slight signs of metallosis were found. Roughening and scratches were observed on the surface of the 12/14 male taper of the modular neck, while the ceramic liner appeared intact, i.e. without evidence of edge fracture. Retrieval analysis was not performed because retrieved components were not made available to our Institute. The fractured femoral head, the ceramic liner and the modular neck were replaced with a ceramic femoral head (Biolox® Delta 40 mm medium neck), a ceramic liner (Biolox® Delta 40 mm) and a modular neck with valgus and increased offset configuration (Modula OZ), respectively (Fig. 7). The patient had a complete functional recovery, and no further complication occurred at a 5.5-year follow-up.

## Discussion

CoC bearings are susceptible to fracture due to brittle nature of ceramic. However, it has been reported that improvements in mechanical properties, also associated with a trend to use larger femoral head, decreased the rate of head fractures. Indeed, the present fracture rate of the fourth-generation ceramic head, calculated equal to 0.007% using 29,495 CoC records of a regional hip register, is lower than the values (range 0.021–1.4%) reported for previous ceramic generations [12, 13] and falls within the range (0.001–0.009%) reported in the literature for the fourth ceramic generation [5, 7].

Common sense suggests that rare events of ceramic head fracture are associated with previous trauma. Evidence supporting this thesis is provided by several reports [14–20]. However, rare fractures of fourth-generation ceramic heads have occurred even in patients without a concurrent or previous remarkable trauma [8–11]. The two cases here described fall in this second category. It has been suggested that ceramic head fracture might be related to edge loading conditions [12]. It is irrefutable that edge loading condition in CoC bearing is mechanically unfavourable, with the severity depending on the magnitude of joint separation [21]. However, edge loading conditions seem to promote formation of wear stripe on head surface, wear area at the rim and, in the worst cases of major joint separation, even small rim chipping in the first contact area due to high peak stresses occurring upon head–rim impact [22–25].

In the present study, the aforementioned damages were neither observed in retrieved components (remark: small rim chipping observed on the retrieved liner was due to impingement of the femoral neck subsequent to head fracture) nor reported in the clinical record of Case 2. Conversely, analysis of retrieved components of Case 1 suggests that ceramic head might fracture under severe in vivo conditions leading to repeated impact load within the bore. Indeed, the fragment appearance of the retrieved femoral head suggests for a two-step mode of failure:

1. Radial vertical microfractures originated at the lower engagement level due to localized high stress areas. Different causes might have contributed to generate the undesired high stress areas, such as angular taper mismatch, incomplete seating, fretting-corrosion of the 12/14 male taper or a combination of these. Although no history of previous falls or remarkable trauma was found in clinical records, it cannot be definitively excluded that the patients had minor trauma. Whatever the root cause is, it seems likely that titanium taper and ceramic bore damage occurred simultaneously, determining progressive increase in the amplitude of the pistoning/rocking micromotion of the head along/about the neck. Neck damage allowed progressive taper shift towards the top of the head bore. This shift changed the loading condition of the ceramic head, stopping the propagation, if any, of the microfractures located at the original lower engagement level;
2. Head catastrophic fracture occurred due to pistoning of the head over the neck due to gross relative motion allowed by damaged 12/14 male taper. Indeed, repeated impacts of the head bore to the taper tip promoted the nucleation of additional radial vertical fatigue fracture that propagated until catastrophic fracture occurred without a concurrent or previous remarkable trauma.

Evidence supporting the proposed mode of failure can be found in the literature. Analysis of retrieved ceramic heads and male tapers has revealed that some minor damage, mainly localized damage to the ridges of the taper machining grooves associated with light metal transfer to the bore surface, takes place in well-working bore–taper junctions [26, 27]. However, suboptimal seating increases both contact pressure and micromotion amplitude at the bore–taper interface under physiological load [28], thus promoting fretting-corrosion damage of the taper [29, 30] and crack nucleation at the ceramic surface [31]. The toughening mechanism of the fourth-generation ceramic is effective in arresting slow crack growth [32]. However, if the damage process within the bore–taper junction proceeds leading to pistoning of the head over the neck, the resulting forceful impacts might determine the transition from quiescent cracks to propagating cracks until they reach a critical size for head fracture. Head fracture through a slow crack growth mechanism has already been supposed by Rankin and co-workers [15]. However, Rankin and co-workers suggested that a previous trauma activated the slow crack growth mechanism, while we speculate that even suboptimal seating of the ceramic head might also trigger the mechanism. Therefore, it is further confirmed the sensitivity of CoC bearings to proper implant positioning and appropriate surgical technique.

There are two other issues that deserve comment:

1. In Case 1, CT images show the presence of gas trapped between ilium and iliopsoas muscle, which disappeared after revision surgery. It cannot be excluded a priori that the generation of gas might be related to products released by the bore–taper interface [33, 34], which had undergone severe damage in the last phase prior to head fracture. However, no specific analyses were performed on the 12/14 male taper to investigate this issue, which deserves further investigation;
2. The two cases here presented involved a modular neck hip prosthesis. At a first glance, it could be speculated an association between fracture of ceramic heads and designs including modular necks. However, it must be highlighted that about 44% of the 29,495 CoC implants have a modular neck design increasing the probability of head fractures in modular neck designs in the analysed records.

## Conclusion

- This study confirms that the fracture of fourth-generation ceramic heads is a rare event;
- The head fracture can occur even without a concurrent or previous remarkable trauma;
- Any increment in micromotion amplitude at the bore–taper junction promotes damage of the interface which might lead to head loosening and, finally, to head fracture;
- Surgeon must continue to pay attention to the assembly of the femoral head: achieving proper head seating on a clean taper is a prerequisite to decrease the risk of occurrence of any damage process within head–taper junction.

## Data Availability

All data generated or analysed during this study are included in this published article.

## Abbreviations

BMI: Body mass index
CoC: Ceramic-on-ceramic
CRP: C-reactive protein
CT-scan: Computed tomography scan
EDX: Energy-dispersive X-ray
ESR: Erythrocyte sedimentation rate
RIPO: Register of Orthopaedic Prosthetic Implant
SEM: Scanning electron microscope
THA: Total hip arthroplasty
